# Bet-hedging as a complex interaction among developmental instability, environmental heterogeneity, dispersal, and life-history strategy

**DOI:** 10.1002/ece3.951

**Published:** 2014-01-23

**Authors:** Samuel M Scheiner

**Affiliations:** Division of Environmental Biology, National Science Foundation4201 Wilson Blvd., Arlington, Virginia, 22230

**Keywords:** Bet-hedging, developmental instability, environmental heterogeneity, model, theory

## Abstract

One potential evolutionary response to environmental heterogeneity is the production of randomly variable offspring through developmental instability, a type of bet-hedging. I used an individual-based, genetically explicit model to examine the evolution of developmental instability. The model considered both temporal and spatial heterogeneity alone and in combination, the effect of migration pattern (stepping stone vs. island), and life-history strategy. I confirmed that temporal heterogeneity alone requires a threshold amount of variation to select for a substantial amount of developmental instability. For spatial heterogeneity only, the response to selection on developmental instability depended on the life-history strategy and the form and pattern of dispersal with the greatest response for island migration when selection occurred before dispersal. Both spatial and temporal variation alone select for similar amounts of instability, but in combination resulted in substantially more instability than either alone. Local adaptation traded off against bet-hedging, but not in a simple linear fashion. I found higher-order interactions between life-history patterns, dispersal rates, dispersal patterns, and environmental heterogeneity that are not explainable by simple intuition. We need additional modeling efforts to understand these interactions and empirical tests that explicitly account for all of these factors.

## Introduction

Central to the theory of evolution is understanding the process of adaptation through trait evolution, including adaptation to varying environments. Historically, varying environments were thought to lead to two possible outcomes: genetic differentiation into multiple genotypes with fixed phenotypes that were each adapted to a different environment, or a single genotype with a fixed phenotype that did sufficiently well in all environments, the jack-of-all-trades strategy. In the 1980s, a mostly neglected third possibility was added to that mix: phenotypic plasticity, whereby a single genotype was able to alter its phenotype in response to the environment so as to match the optimum in each specific environment (Sarkar 2004). At the same time, another potential solution was being proposed: the production of phenotypically variable offspring through some sort of random process, rather than through a process of direct environmental responsiveness (Slatkin 1974; Philippi and Seger 1989; Simons and Johnston 1997; Starrfelt and Kokko 2012). This process has been given a variety of names such as adaptive coin flipping, developmental noise, and diversified bet-hedging. In this study, I will refer to the process of producing randomly variable offspring as developmental instability, and the selective process as bet-hedging. Somewhat confusingly, the jack-of-all-trades strategy is sometimes referred to as conservative bet-hedging (Starrfelt and Kokko 2012), a usage that I will avoid.

For the past 30 years, phenotypic plasticity and developmental instability mostly have been dealt with independently, both with regard to theory and empirical study. Yet both are alternative outcomes to selection in a varying environment and might interact with each other. This paper is the second of a set of three examining the intersection of phenotypic plasticity, developmental instability, environmental variation, and life-history strategy. The previous paper (Scheiner 2013) examined phenotypic plasticity alone and explored the many different ways that environmental variation could be created by the mode and pattern of spatial and temporal heterogeneity in combination with the mode and pattern of movement. This paper does the same for developmental instability alone. The third paper will explore the intersection of plasticity and instability.

Developmental instability has been shown to have a genetic basis (e.g., Scheiner et al. 1991; Ros et al. 2004; Ibáñez-Escriche et al. 2008; Shen et al. 2012; Tonsor et al. 2013) and thus can be selected for. As an adaptive response to environmental heterogeneity, developmental instability maximizes the fitness of a lineage by which increasing the phenotypic variation among individuals of that lineage (Starrfelt and Kokko 2012). In a uniform environment with stabilizing selection, matching the optimal phenotype will always maximize fitness so that selection will minimize the phenotypic variance. In a heterogeneous environment, bet-hedging is favored when an increase in the phenotypic variance means that at least some individuals will express the phenotype with the maximal fitness. Or as Starrfelt and Kokko (2012) explain it, for a given set of individuals all with the same genotype, there is a trade-off between their variance in fitness and the correlation of their fitnesses. Processes that reduce the correlation among individuals include dispersal in space or time and increasing their phenotypic variance. One question explored in this study is how those processes play out against each other.

Previous models of bet-hedging fall into two categories: optimality models (Cooper and Kaplan 1982; McNamara et al. 1995; Donaldson-Matasci et al. 2008; Marshall et al. 2008; Rajon et al. 2009) and quantitative genetic models (Slatkin and Lande 1976; Bull 1987; Sasaki and Ellner 1995). Nearly all focus on temporal environmental heterogeneity, the exceptions being Marshall et al. (2008) and Rajon et al. (2009). None examined the interaction between temporal and spatial heterogeneity. The models differed as to whether they treated environmental or trait variation as discrete (almost always dichotomous) or continuous. As summarized by Starrfelt and Kokko (2012), in these models, temporal heterogeneity was more likely to favor bet-hedging than spatial heterogeneity. In the bet-hedging literature, temporal heterogeneity and spatial heterogeneity are referred to as coarse grained and fine grained, respectively. In most previous bet-hedging models, spatial heterogeneity was considered to be fine grained because it was assumed that all lineages would disperse to all environments in proportion with the frequency of those environments, that is, a propagule pool.

### My model

My model is an individual-based simulation with explicit genes, thus differing from all previous modeling approaches to bet-hedging, although being most closely related to the quantitative genetic models. In my model, the genotype consists of two classes of loci, those with a deterministic phenotypic expression and those that allow for random deviation from that phenotype. This genetic architecture allows for local adaptation and developmental instability to evolve independently. I am not aware of any studies that have directly addressed this genetic assumption, but it seems reasonable that the mean and the variance of a trait would be genetically independent in at least some species for some range of trait values.

I consider three patterns of environmental heterogeneity: (1) temporal variation alone, (2) spatial variation alone, and (3) both temporal and spatial variation. When temporal variation occurs, it can be independent from one generation to the next or it can be autocorrelated (ranging from −75% to 75%). When spatial variation occurs, it is among demes along a linear gradient. When temporal and spatial variation both occur, the pattern of temporal variation can differ among demes or be the same for all demes.

I treat environmental heterogeneity differently from previous efforts in ways that, as I will show, can have profound effects on evolutionary outcomes. This difference comes from the interaction of three factors: the pattern of environmental heterogeneity, the pattern of dispersal, and the timing of dispersal. In my model, in contrast to most previous bet-hedging models, space has structure (a set of equal size demes arranged in a line) and movement among environments occurs through larval/adult dispersal, rather than as a propagule pool. I consider two different movement patterns: stepping-stone migration and island migration. For stepping-stone migration, movement occurs among nearby demes with the probability of movement decreasing with distance. For island migration, movement occurs among all demes with equal probability, although more complex movement rules are possible. Island migration is equivalent to a propagule pool in distance moved, although the latter assumes 100% dispersal, while my model considers a range of dispersal rates. In previous models, all lineages experienced the complete range of environmental variation in a single generation, while in my model, a given lineage would experience a more limited amount of that total environmental heterogeneity in one or a few generations.

Finally, my model considers two different life-history strategies defined by the timing of dispersal, before selection and after selection. The propagule pool assumption of previous models is equivalent to movement before selection. This difference in the timing of movement affects whether a given set of siblings experience the same or different selective environments. In other words, the timing of dispersal can also change the extent to which a lineage perceives the environment as coarse grained or fine grained.

## Model Structure

My individual-based simulation (summary of parameters in Table 1) used a gene-based model of adaptation to environmental heterogeneity. That heterogeneity could occur in two ways: a single deme with temporal variation or multiple demes arrayed along an environmental gradient. In the latter case, I considered both spatial heterogeneity alone and in combination with temporal heterogeneity. The gradient consisted of a linear change in the optimal phenotype. Local adaptation could occur through genetic differentiation of deterministically expressed genes with partial adaption possible. Developmental instability was due to genes that determined the variance of a random deviate from the deterministic phenotype.

**Table 1:** Summary of the model parameters.

Fixed parameters
Number of deterministic and developmental instability loci = 5 each
Steepness of the gradient (change in optimum in adjacent demes) = 0.4 units
Strength of selection within demes (*σ*) = 2 units
Number of generations = 10,000
Parameters explored
Length of the environmental gradient: 1 or 50 demes
Population size: 1000 or 100 individuals/deme
Life-history pattern: selection before dispersal versus dispersal before selection
Dispersal pattern: stepping stone or island
Dispersal rate (5–84%)
Magnitude of environmental change (0–50% of the length of the spatial gradient)
Correlation of environmental change within and among generations (−0.75 to 0.75)

The model was implemented in Fortran 77 (the computer code is available from Dryad, doi:10.5061/dryad.9bp88). The metapopulation consisted of a linear array of 50 demes. An environmental gradient was created by varying the optimal value of a single trait (phenotype) in a linear fashion along the array from −9.8 to +9.8 arbitrary units at the ends of the gradient, that is, the optimal phenotype in adjacent demes differed by 0.4 units. An individual's phenotype (trait value) was determined by 10 diploid loci: five deterministic loci and five instability loci. The deterministic loci contributed additively to the trait. The phenotype of each individual was:


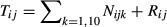
1

where *T*_*ij*_ is the phenotype of the *j*th individual that develops in the *i*th environment (deme 1–50) and *N*_*ijk*_ is the allelic value of the *k*th deterministic allele of that individual. *R*_*ij*_ is the developmental instability of that individual which was determined by a Gaussian normal deviate with a standard deviation equal to 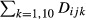, where *D*_*ijk*_ is the allelic value of the *k*th instability allele.

Life-history events occurred in one of two sequences: (1) birth, followed by development (i.e., the phase in the life cycle when the phenotype is determined), then dispersal, selection, and reproduction (denoted as “move first”); or alternatively, (2) birth, development, selection, dispersal, and then reproduction (denote as “select first”). Selection was based on survival with the probability of surviving being a Gaussian function of the difference between an individual's phenotype and the locally optimal phenotype. Fitness (the probability of surviving) was determined as:


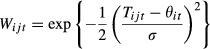
2

where *W*_*itj*_ is the fitness of the *j*th individual undergoing selection in the *i*th environment in the *t*th generation, *T*_*itj*_ is the phenotype of that individual, *θ*_*it*_ is the optimal phenotype in that environment, *σ* is the strength of selection (selection weakens as *σ* increases). For all simulations, *σ *= 2; the length of the spatial gradient across demes was approximately 2.5 standard deviations (*σ*) of the width of the within-deme selection function. In other words, an individual whose phenotype perfectly matched its environment had a probability of survival of 100%; moving that individual 20 demes (equivalent to 1 standard deviation of the width of selection) would reduce its probability of survival approximately 22%.

If the environment varied over time, that change occurred once at the beginning of each generation. All demes could vary in a given generation and that variation could be independent among demes or be synchronized among demes. In the latter case, the optimal phenotype in all demes changed by the same magnitude and direction. That variation could be uncorrelated from one generation to the next or be correlated among generations. If variation was independent among demes, each deme had its own pattern of temporal autocorrelation. Temporal autocorrelation was simulated using the recursion:


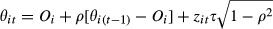
3

where *θ*_*it*_ is the environment at either development or selection in the *i*th deme in generation *t*, *O*_*i*_ is the mean or fixed environment in the *i*th deme (a linear function of *i*), *τ* is the standard deviation of environmental variation, *ρ* is the temporal autocorrelation coefficient, and *z*_*it*_ is a sequence of independent zero-mean, unit-variance Gaussian random deviates. For simulations without temporal variation, *τ *= 0, and for uncorrelated temporal variation, *ρ *= 0. The standard deviation of environmental noise (*τ*) is shown as a percentage of the difference in the optima at the two ends of the gradient. The autocorrelation (*ρ*) varied from −75% to 75%.

**Figure 1:**
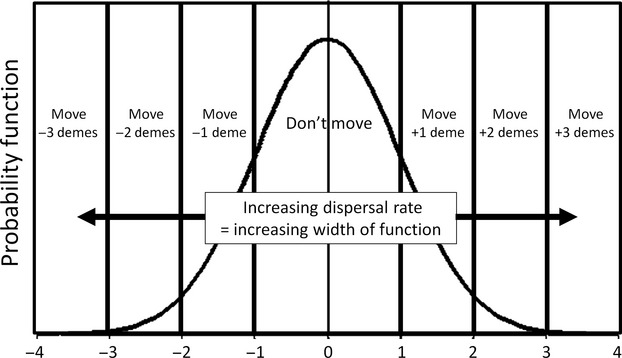
Probability density function for the random variable determining the likelihood that an individual would move between demes and the distance of that movement. Shown is a dispersal probability of 32%. An increase or decrease in that probability is equivalent to increasing or decreasing the width of the function. (from Scheiner and Holt 2012).

Dispersal occurred in one of two patterns: stepping stone or island. For the stepping-stone migration pattern, the dispersal probability and the distance moved were determined using a zero-mean Gaussian random number, so that the probability of moving and the average distance moved were correlated (Fig. 1). Increasing the dispersal probability was made by increasing the variance of the Gaussian so that both more individuals were likely to move, and they were likely to move farther. Individuals that would otherwise disperse beyond the end of the gradient moved to the terminal demes. For the island migration pattern, each individual had a fixed probability of moving. If it moved, it had an equal probability of moving to any of the other demes. For both patterns, dispersal per se had no cost; survival during dispersal was 100%. The dispersal probabilities were the same for the two life-history strategies: *move first* and *select first*; however, the absolute number of individuals dispersing was fewer for the *select first* life-history strategy because of reductions in deme sizes due to selection.

Reproduction was accomplished by assembling pairs of individuals within a deme at random with replacement (allowing for self-fertilization), with each pair producing one offspring, then repeating until the carrying capacity of that deme was reached. This procedure assumes soft selection, in that local population size was determined independently of the outcome of survival selection and results in individuals competing for offspring. The model assumes that the spatial scale of reproduction and mating matches that of density dependence and the grain of the selective environment.

Each simulation was initialized with 100 individuals being born in each deme, or 1000 individuals for simulations with just temporal variation in a single deme. Those deme and total population sizes (5000 individuals (50 × 100) and 1000 individuals, respectively) were chosen to minimize genetic drift and the effects of population size on among-individual fitness variance (Starrfelt and Kokko 2012), while making the simulations computationally tractable. For each individual in the initial generation, deterministic allelic values were chosen independently from the values −2, −1, 0, 1, and 2, with each value being equally likely (even though initial values are discrete, due to mutation, allelic values are continuous variables after the initial generation). When new offspring were generated, each allele mutated with a probability of 10%. [Lower mutation rates mainly changed the time-scale over which evolution occurs, rather than the eventual outcome (Scheiner and Holt 2012).] When a mutation occurred, the allelic value was changed by adding a Gaussian deviate (mean of zero and a standard deviation of 0.1 units) to the previous allelic value (i.e., this was an infinite-alleles model). For the developmental instability loci, all alleles began with a value of zero. The probability of mutation and the standard deviation of the Gaussian deviate were the same as the other loci, but only positive values were retained, negative values were set to zero.

All simulations were run for 10,000 generations to ensure that the equilibrium point (the point after which all calculated quantities showed no further directional trend) was reached. Each parameter combination was replicated 20 times, and the results shown are the means of those replicates. The coefficients of variation in reported parameters were generally low (1–5%). If the metapopulation went extinct, additional realizations were run until 20 successful replications were achieved; for some parameter combinations (see Results), the extinction probability was 100% (i.e., no successful replications in 60 runs). Reported outcomes were averaged over successful replications only.

## Results

### Temporal heterogeneity alone

**Figure 2:**
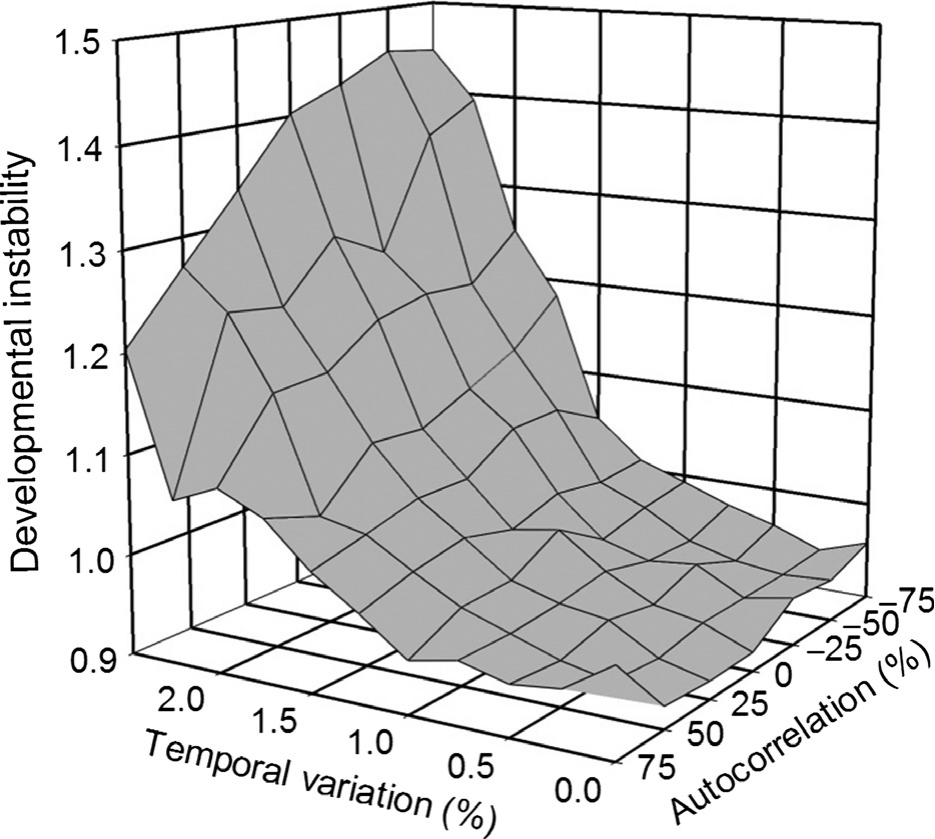
The effect of temporal variation on selection for developmental instability (mean *R*_*ij*_). Variation is scaled relative to the strength of selection (*σ*).

When the only environmental heterogeneity was temporal and the amount of variation was less than the width (*σ*) of the selection function, there was little increase in the amount of developmental instability (mean *R*_*ij*_) as environmental heterogeneity increased (Fig. 2). Below that value, developmental instability is at its selection-mutation balance, approximately 0.93 for the parameter values used here. Beyond that point, instability increased substantially with temporal heterogeneity. The response was smaller when the environment was positively autocorrelated because individuals tended to experience the same environment from one generation to the next. The increase in developmental instability was accompanied by a decrease in the mean fitness of the population (results not shown). Thus, temporal variation selected for increased developmental instability resulting in bet-hedging through a trade-off between mean fitness and phenotypic variance.

### Spatial heterogeneity alone

**Figure 3:**
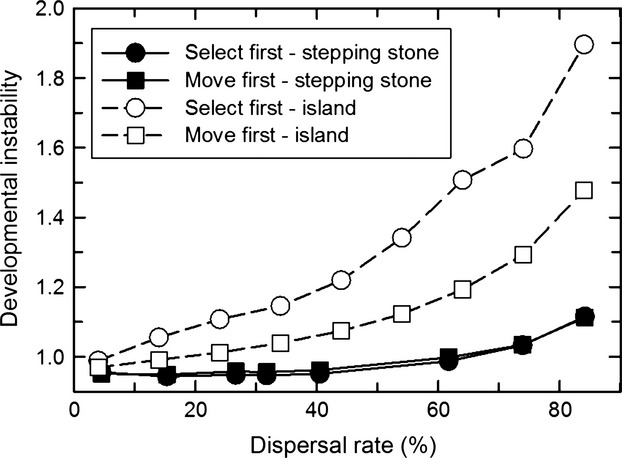
The effect of spatial variation on selection for developmental instability (mean *R*_*ij*_), showing the effects of the two dispersal patterns and life-history strategies.

When heterogeneity was spatial only, the response to selection on developmental instability depended on the life-history strategy and the form and pattern of dispersal. In all instances, instability (mean *R*_*ij*_) increased at greater dispersal rates (Fig. 3). For the stepping-stone migration pattern, the response of instability was small and did not depend on the life-history strategy.

For the island migration pattern, the response to selection was greater overall and similar in magnitude to that of temporal heterogeneity. With island migration, individuals could disperse across the entire environmental gradient, so that a lineage experienced greater heterogeneity. In contrast to the stepping-stone migration pattern, for the island pattern, the timing of dispersal mattered with a greater response when selection occurred before dispersal. Thus, both temporal and spatial heterogeneity promoted bet-hedging. Again, the increase in developmental instability was accompanied by a decrease in the mean fitness of the population.

### Combining temporal and spatial heterogeneity

**Figure 4:**
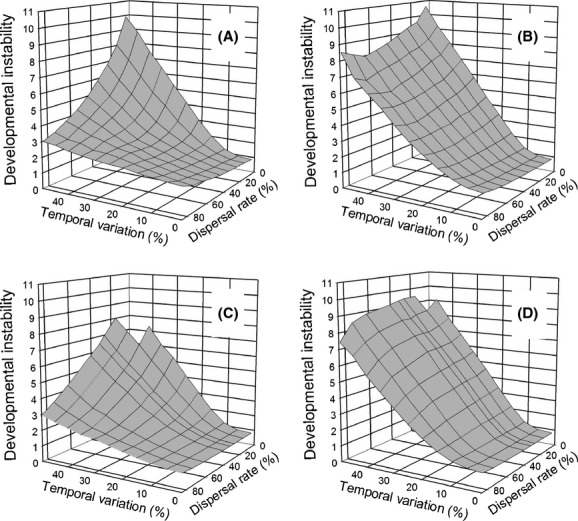
The effect of the interaction of dispersal rate and temporal variation on the propensity for developmental instability (mean *R*_*ij*_) to be favored by selection when temporal variation is independent among demes (correlation among generations = 0). (A) and (B) Dispersal by the island migration pattern; (C) and (D) dispersal by the stepping-stone migration pattern. (A) and (C) Selection before dispersal (*select first*); (B) and (D) dispersal before selection (*move first*).

**Figure 5:**
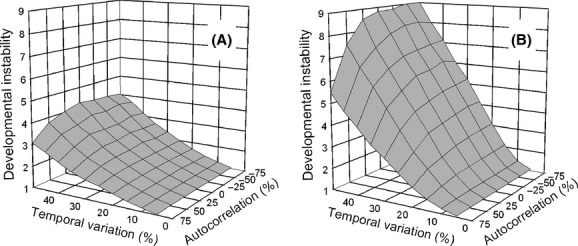
The effect of the interaction of temporal variation and among-generation correlation on the propensity for developmental instability (mean *R*_*ij*_) to be favored by selection. Dispersal was by the stepping-stone migration pattern (dispersal rate = 64%). Temporal variation is scaled as a percentage of the length of the environmental gradient and is independent among demes. (A) Selection before dispersal (*select first*); (B) dispersal before selection (*move first*).

When temporal heterogeneity was combined with spatial heterogeneity, the two factors resulted in a large selective response of developmental instability (mean *R*_*ij*_). Increasing temporal heterogeneity that was not synchronized among demes, selected for increasing developmental instability for both life-history strategies (Fig. 4). Similar to the pattern for spatial heterogeneity alone, the island migration pattern selected for greater instability than the stepping-stone migration pattern, especially at low dispersal rates and high amounts of temporal heterogeneity. Contrary to the pattern for spatial heterogeneity alone, however, the *move first* life-history strategy selected for greater instability than the *select first* life-history strategy, especially at high dispersal rates and high amounts of temporal heterogeneity. Although increases in both dispersal and temporal heterogeneity increased the environmental variation experienced by a lineage, for the *select first* life-history strategy, they had a negative synergy. As with temporal heterogeneity alone, positive autocorrelation selected for lower amounts of instability, especially for the *move first* life-history pattern when temporal variation was high (Fig. 5).

**Figure 6:**
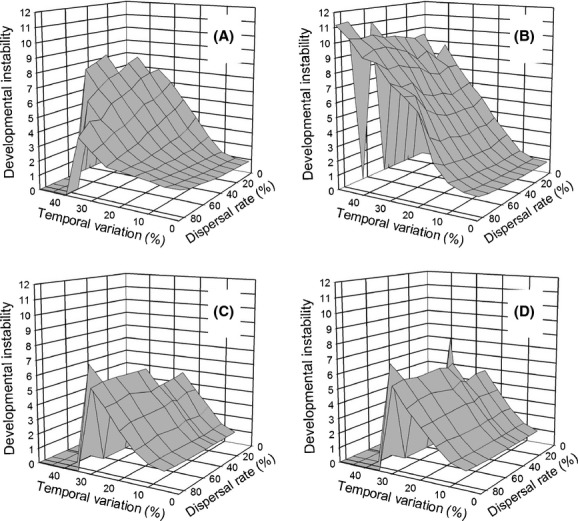
The effect of the interaction of dispersal rate and temporal variation on the propensity for developmental instability (mean *R*_*ij*_) to be favored by selection when temporal variation is synchronized among demes (correlation among generations = 0). (A) and (B) Dispersal by the island migration pattern; (C) and (D) dispersal by the stepping-stone migration pattern. (A) and (C) Selection before dispersal (*select first*); (B) and (D) dispersal before selection (*move first*).

If temporal changes were synchronized among the demes, selection for instability was greater overall (Fig. 6). For the island migration pattern, the *move first* migration pattern resulted in a greater response than the *select first* migration pattern, as when variation within demes was independent. However, in this case, the greatest response was when both the dispersal rate and temporal heterogeneity were large. In contrast, for the stepping-stone migration pattern, the two life-history strategies evinced similar amounts of instability. For all of these simulations (Figs 4, 6), the zero values for instability when temporal heterogeneity was very high (e.g., the “notch” in the upper right-hand corner of Fig. 4C, D) were due to extinction of the metapopulation rather than a lack of an evolutionary response.

**Figure 7:**
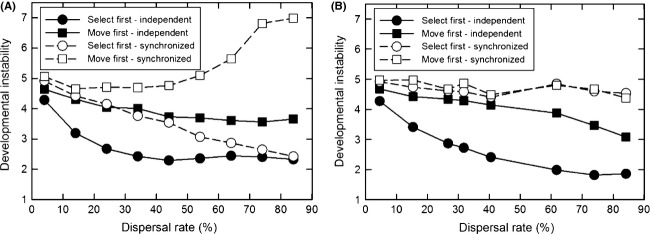
The effect of dispersal rate on the propensity for developmental instability (mean *R*_*ij*_) to be favored by selection when there is both temporal and spatial environmental heterogeneity. The standard deviation of the temporal variation was 25% of the length of the spatial gradient (correlation among generations = 0). Temporal variation was either independent among demes (solid symbols) or synchronized among demes (open symbols). Dispersal occurred either after selection (*select first*, circle) or before selection (*move first*, square). (A) Dispersal by the island migration pattern; (B) dispersal by the stepping-stone migration pattern.

To better see the combined effects of the pattern of temporal heterogeneity, dispersal pattern and rate, and life-history strategy, Fig. 7 shows a slice through Figs. 4, 6 at 25% temporal variation. Synchronized temporal variation always selected for greater instability than independent variation. However, there is a complex interaction between the life-history strategy, the migration pattern, and the dispersal rate. At this amount of temporal variation, when it was synchronized among demes, for the *select first* life-history strategy, higher dispersal rates selected for less instability with island migration as compared to stepping-stone migration, but that was reversed for the *move first* life-history strategy.

### Local adaptation

**Figure 8:**
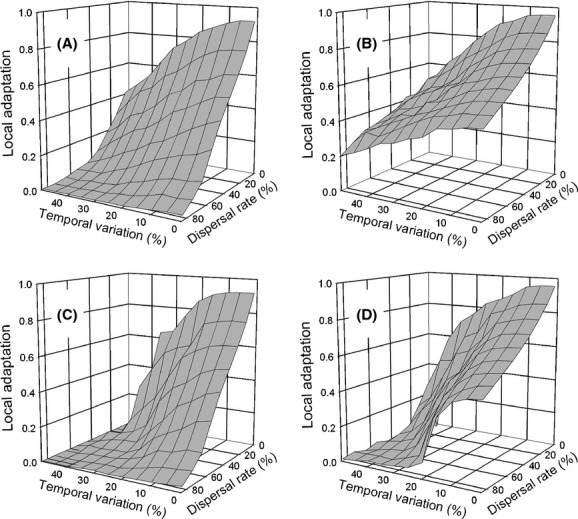
The effect of the interaction of dispersal rate and temporal variation on the propensity for local adaptation (mean *T*_*ij*_; dispersal by the island migration pattern, correlation among generations = 0). (A) and (B) Temporal variation within each deme is independent; (C) and (D) temporal variation within each deme is synchronized. (A) and (C) Selection before dispersal (*select first*); (B) and (D) dispersal before selection (*move first*).

Local adaptation is measured by the mean phenotype of the metapopulation (*T*_*ij*_). In Fig. 8, a value of one for local adaptation indicates that the mean phenotype of each deme exactly matched its selective optimum; a value of 0 indicates that the mean phenotype of all demes was equal (i.e., no genetic differentiation). Developmental instability affects the amount of variation around that mean, and subsequently, the mean fitness of the population, but does not affect the mean phenotype and selection for local adaptation (based on simulations in which developmental instability was absent, results not shown). For the island migration pattern, as dispersal rate and the amount of temporal variation increased, genetic differentiation decreased, as expected. This effect was greater for the *select first* life-history strategy. However, it was not affected by whether the temporal variation was synchronized among demes (compare Fig. 8A, C). In contrast, for the *move first* life-history strategy, genetic differentiation decreased less as dispersal rate increased when temporal variation was low (compare Fig. 8A, B) and synchronization decreased differentiation when temporal variation was high (compare Fig. 8B, D). The 0 values in Fig. 8C, D are because the metapopulation went extinct. Developmental instability as a mode of bet-hedging was more strongly selected for as local adaptation was weakened (compare Figs. 8A, 4A; 8B, 4B; 8C, 6A; 8D, 6B). In contrast, for the stepping-stone migration pattern, local adaptation was little affected by dispersal rate and the amount of temporal variation (Fig. S1). Thus, local adaptation traded off against bet-hedging, but not in a simple linear fashion.

## Discussion

### The expected

My results are a combination of the expected, the unexpected, and the yet to be explained. Expected results are of two sorts, those predicted by previous models and those not explored by previous models but expected by intuition. Quantitative genetic models (Slatkin and Lande 1976; Bull 1987; Sasaki and Ellner 1995) predict that for temporal heterogeneity alone, developmental instability would be selected for only above a threshold related to the width of the selection function. I found that instability increased substantially above that value, but that there was some response even below that threshold, possibly because of the finite, stochastic nature of my simulations as compared to the infinite population assumed in the quantitative genetic models.

For spatial heterogeneity alone, the effects of dispersal rate and dispersal pattern were those expected based on intuition; greater developmental instability was selected for with higher dispersal rates and with an island migration pattern as compared to a stepping-stone pattern (Fig. 3). For stepping-stone migration, because the distance moved is tied to the probability of dispersing, the average distance moved is low except for very high dispersal rates. For a 74% dispersal rate, the mean dispersal distance is 2.9 demes and 99% of individuals disperse four demes or fewer. Because dispersal occurs in both directions, 99% of offspring are born within nine demes of their parents' birth deme (their parent's deme +4 to either side). For an 84% probability of dispersal, the mean dispersal distance is 4.5 demes, although there is a long tail such that 4% of individuals move nine demes or more. The total gradient of 50 demes has a change in the optimal phenotype of only 2.5 standard deviations of the width of the selection function, so that even with an 84% probability of moving, most individuals are in demes with a change in the optimal phenotype of only 0.22 standard deviations of their parents' optimum. In contrast, for island migration, an individual at the center of the gradient will move 12.5 demes on average (a quarter the length of the gradient), and those at the ends of the gradient will move 25 demes on average, or approximately 0.63 and 1.25 standard deviations, respectively.

The effect of temporal autocorrelation was, as one would intuit, to select for reduced instability with positive autocorrelation, especially for the *move first* life-history strategy (Figs. 2, 5). This decrease occurs because a positive autocorrelation reduces the environmental variation experienced by a lineage over a given stretch of time. These results contradict the assertion of Sasaki and Ellner (1995) that there should be no effect of autocorrelation. However, that assertion is not based on an analytic result. Slatkin and Lande (1976) claim that a positive autocorrelation should lower the threshold amount of temporal variation necessary to select for instability and negative autocorrelation should raise the threshold. Such a change in threshold is not apparent in Fig. 2, if anything the trend is in the opposite direction; however, the data are noisy, and a finer-scale examination with many more replicates might detect it.

### The unexpected

Unexpected results are those that either contradict previous models or were not predicted by simple intuition. The greatest unexpected result was the strong non-additive synergy between temporal and spatial heterogeneity resulting in much greater instability in combination (compare Figs. 2, 3 with Fig. 4). For low to intermediate dispersal rates and high amounts of temporal heterogeneity, developmental instability was five to ten times as great than for temporal or spatial heterogeneity alone.

This effect was particularly striking for stepping-stone migration. This result was unexpected given that for spatial variation alone at intermediate dispersal rates, stepping-stone migration had substantially lower amounts of instability as compared with island migration. In hindsight, I can explain these similar amounts of instability with high temporal variation. In Fig. 4, temporal variation is scaled relative to the length of the gradient so that at high temporal variation, individuals that do not move are still likely to be subject to a change in the optimal phenotype equivalent of moving 25 demes, the same as experienced with island migration.

Rajon et al. (2009) using a two patch model and dichotomous phenotypes concluded that greater bet-hedging would be selected for with a lower dispersal rate. I did not find that result for spatial variation alone, but did find greater instability favored when spatial heterogeneity was combined with high temporal heterogeneity and the *select first* life-history strategy. The Rajon et al. (2009) model is structured somewhat differently, but it also has life events ordered similar to the *select first* life-history strategy: reproduction then selection then dispersal.

I also did not expect that synchronizing temporal change among demes would more strongly favor instability (Fig. 6). In hindsight, this result makes sense once the effect is considered as an outcome of demography and not just selection. A very large shift in the optimum phenotype between generations is likely to result in very high mortality and even complete extinction of all individuals in a deme. Developmental instability hedges against this outcome by making it more likely that at least a few individuals will survive, what Starrfelt and Kokko (2012) refer to as implicit frequency dependence. With synchronization, these large shifts occur in all demes together, thus selecting simultaneously for instability across the entire metapopulation. Because this result is partially a function of population demography, it was not explored in any of the previous models.

Also in hindsight, it is clear why for stepping-stone migration, synchronization eliminated the synergy between temporal and spatial heterogeneity. For stepping-stone migration, spatial heterogeneity alone showed very little effect of dispersal rate (Fig. 2) and that lack of effect holds with the addition of temporal heterogeneity (Figs. 6, 7). With stepping-stone migration, individuals move short distances. When temporal variation is unsynchronized among demes, two adjacent demes could have very different optimal phenotypes in a given generation so that it would be as if the individual had moved a large distance. Synchronization eliminates that effect.

Starrfelt and Kokko (2012) concluded that temporal heterogeneity (a coarse-grained environment in their terminology) is more likely to select for bet-hedging. However, I found that both temporal and spatial heterogeneity selected for similar amounts of developmental instability, at least for island migration (Figs. 2, 3), which is the migration pattern most consistent with the models that they considered. However, they note (without providing details) that this conclusion could change if competition occurs within demes of a spatially heterogeneous environment because it increases the variance in fitness experienced by individuals of the same lineage in different demes. In my model, individuals compete within demes for offspring, possibly explaining why I did not find a difference between temporal and spatial heterogeneity.

### The yet to be explained

Still unexplained are the higher-order interactions apparent between life-history pattern, dispersal rate, dispersal pattern, and spatial and temporal heterogeneity. Despite my own attempts, and discussions with several colleagues, I am unable to come up with intuitive answers to these results. I call upon you, the reader, to help supply such explanations. These anomalies also call for new analytic models that might provide explanations.

When there is only spatial heterogeneity, the *select first* life-history pattern with island migration and high dispersal resulted in the greatest amount of instability (Fig. 3). That result by itself calls for an explanation. Intuition says that the *move first* life-history pattern should select for greater instability because the individuals within a single generation of a given lineage experience a broad range of selective environments. In contrast, under the *select first* life-history pattern, those individuals all experience a single selective environment within a generation and variable selective environments across generations. That pattern should select for less instability. Yet, the opposite pattern was seen.

In contrast when temporal heterogeneity was high, the *select first* life-history pattern resulted in substantially lower amounts of instability than the *move first* life-history strategy, but only at high dispersal rates (Fig. 4). So intuition holds, but only for a limited part of the parameter space where a given lineage experiences large both within- and among-generation environmental heterogeneity.

At high levels of temporal heterogeneity, the *move first* life-history pattern showed unexpected interactions between migration pattern and temporal synchronicity (Figs. 4B, 4D, 7B). The high-order nature of these patterns makes it difficult to come up with explanations. These unexplained patterns cannot be due to problems with the software code as they only appear for particular combinations of parameter values and processes, and not for either whole sections of code or code combinations.

## Conclusions

From these simulations, we learned three new things. First, I confirmed that temporal heterogeneity requires a threshold amount of variation to select for a substantial amount of developmental instability in a quantitative trait (Slatkin and Lande 1976; Sasaki and Ellner 1995). Second, the claim that greater bet-hedging would be selected for at lower dispersal rates (Rajon et al. 2009) holds only when there is also substantial temporal heterogeneity. Contrary to previous claims (Starrfelt and Kokko 2012), both spatial and temporal variations select for similar amounts of instability. Third, when temporal variation is synchronized across space (e.g., along an elevational gradient), instability is favored because it reduces extinction risk. Fourth, temporal and spatial heterogeneity can act in synergy to select for much higher amounts of instability than either alone. In addition, I found higher-order interactions between life-history patterns, dispersal rates, dispersal patterns, and environmental heterogeneity.

Several next steps are called for. First is the need for a constitutive theory of the evolution of bet-hedging that would unite the various types and flavors of models into an explicit conceptual framework. [See Scheiner (2010) and Scheiner and Willig (2011) for a description of constitutive theories and Scheiner (2013) for a recent example for the evolution of phenotypic plasticity.] Second is the need for additional modeling. The results of my simulations differ from some previous models, but so does the structure of my model. Most previous models considered either dichotomous environments or trait values or both and used a propagule pool dispersal pattern. Of particular need are analytic quantitative genetic models of spatial heterogeneity alone and in combination with temporal heterogeneity. Third is the need for empirical tests of these models. While anecdotal data exist to show that bet-hedging strategies exist, I am not aware of any explicit test of any bet-hedging model. Such tests will likely require building more specific models. More important, we need to identify what theoretical aspects are in critical need of testing. Such identification comes from the development of an explicit constitutive theory.

Starrfelt and Kokko (2012) point out that environments can have both temporal and spatial heterogeneity, which they term medium grained. I suggest that we abandon the fine-grained/coarse-grained terminology altogether as it reinforces a false dichotomy in our thinking. That terminology refers to what happens in a single generation. But selection on bet-hedging is the result of the range of environments experienced by a lineage over multiple generations. “Fine grained” is typically thought of as any genotype experiencing the entire range of environments within a single generation and “coarse grained” as experiencing just a single environment, although this depends on dispersal distances relative to the grain of spatial heterogeneity and generation time relative to the grain of temporal heterogeneity. When there are both spatial variation and temporal variation and movement is viscous, a lineage may experience only some of that variation within a single or limited number of generations. So rather than a dichotomy, they are ends of a spectrum that, as shown here, can result in unexpected behaviors under intermediate conditions.

Previous models examined just one or two patterns of spatial or temporal heterogeneity, dispersal patterns, or life-history strategies. The complex results found here lead me to reiterate my previous statement (Scheiner 2013) about the importance of knowing the ecological and life-history context of trait instability. As with plasticity, to understand selection on developmental instability requires studying the interactions among all of these components and making that complete context explicit in any empirical test. Developmental instability and phenotypic plasticity are alternative evolutionary responses to environmental heterogeneity. The next study in this set will examine what happens when both are potential outcomes.

## Data Accessibility

The program code is archived with Dryad. Data available from the Dryad Digital Repository: http://doi.org/10.5061/dryad.9bp88

## Supporting Information

Figure S1 The effect of the interaction of dispersal rate and temporal variation on the propensity for local adaptation (mean *T^ij^* dispersal by the stepping-stone migration pattern, correlation among generations = 0). (A) and (B) Temporal variation within each deme is independent; (C) and (D) temporal variation within each deme is synchronized. (A) and (C) Selection before dispersal (*select first*); (B) and (D) dispersal before selection (*move first*).
