# Supplementary material for: Bet-hedging as a complex interaction among developmental instability, environmental heterogeneity, dispersal, and life-history strategy
Source: Ecol Evol. 2014 Jan 23;4(4):505–15. doi: 10.1002/ece3.951 (PMC3936396; doi:10.1002/ece3.951)
Supplement: Figure S1 — The effect of the interaction of dispersal rate and temporal variation on the propensity for local adaptation (mean Tij dispersal by the stepping-stone migration pattern, correlation among generations = 0). (A) and (B) Temporal variation within each deme is independent; (C) and (D) temporal variation within each deme is synchronized. (A) and (C) Selection before dispersal (select first); (B) and (D) dispersal before selection (move first). [file ece30004-0505-sd1.pdf]

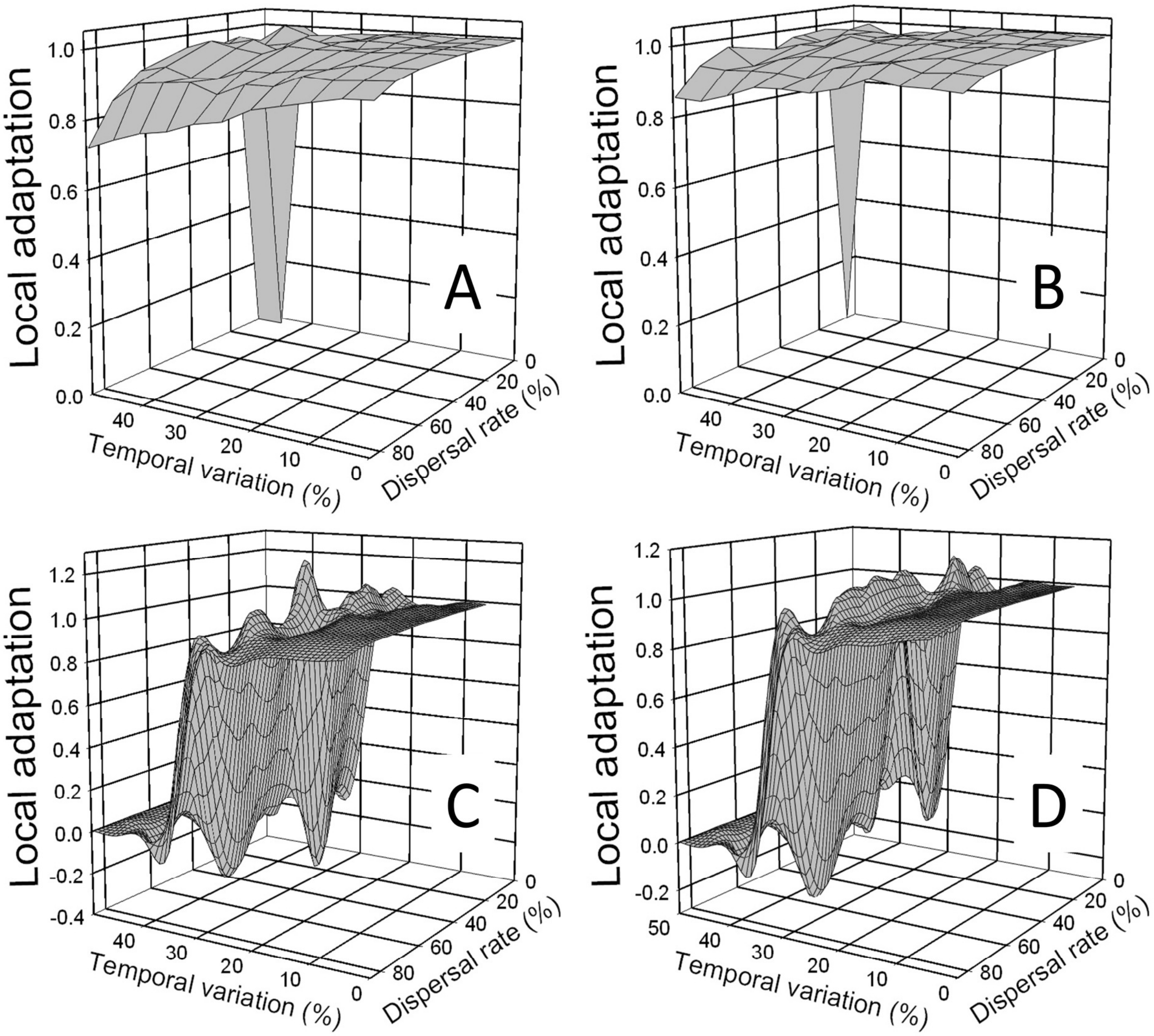

Figure S2. The effect of the interaction of dispersal rate and temporal variation on the propensity for local adaptation (mean T_{ij}; dispersal by the stepping stone migration pattern, correlation among generations = 0). (A) and (B) Temporal variation within each deme is independent; (C) and (D) temporal variation within each deme is synchronized. (A) and (C) Selection before dispersal (*select first*); (B) and (D) dispersal before selection (*move first*).
